# The Brief Emotion Dysregulation Scale: Development, Preliminary Validation, and Recommendations for Use

**DOI:** 10.1177/10731911231161800

**Published:** 2023-03-24

**Authors:** Andrea M. Wycoff, Sarah A. Griffin, Ashley C. Helle, Alison M. Haney, Ashley L. Watts, Timothy J. Trull

**Affiliations:** 1 Brown University, Providence, RI, USA; 2 University of Houston–Clear Lake, TX, USA; 3 University of Missouri, Columbia, USA

**Keywords:** emotion dysregulation, emotion regulation, affective instability, transdiagnostic, scale development

## Abstract

Emotion dysregulation is a multi-faceted, transdiagnostic construct, and its assessment is crucial for characterizing its role in the development, maintenance, and treatment of psychiatric problems. We developed the Brief Emotion Dysregulation Scale (BEDS) to capture four components of emotion dysregulation: *sensitivity*, *lability*, *reactivity*, and *consequences*. We examined factor structure and construct validity in four independent samples of college students (*N* = 1,485). We elected to treat *consequences* as a separate index of problems associated with emotion dysregulation. Exploratory and confirmatory factor analyses did not support the reactivity subscale and instead supported a well-fitting two-factor solution for sensitivity and lability. Multi-group analyses demonstrated strong factorial invariance by gender. The resulting 12-item BEDS includes *sensitivity* and *lability* subscales and a separate *consequences* scale to indicate associated problems. Convergent correlations suggested good construct validity. This provides preliminary support for the BEDS as a brief transdiagnostic screening tool for emotion dysregulation and associated consequences.

The process of emotion dysregulation has been conceptualized as consisting of four components: sensitivity to emotional stimuli, heightened and labile negative affect, a combination of inadequate appropriate emotion regulation strategies and increased maladaptive emotion regulation strategies, and consequences of emotion dysregulation (Carpenter & Trull, 2013). These four components describe the processes by which emotion dysregulation occurs and is maintained over time. Although much of the research on emotion dysregulation occurs in the context of borderline personality disorder (BPD; Carpenter & Trull, 2013; Crowell et al., 2009; Linehan, 1993), emotion dysregulation is associated with a range of psychopathology, including generalized anxiety disorder (Mennin et al., 2005), attention deficit/hyperactivity disorder (Musser & Nigg, 2019; Richard-Lepouriel et al., 2016; Shaw et al., 2014), depressive and bipolar disorders (Fowler et al., 2016), and bulimia nervosa (Gordon et al., 2016), among others. Furthermore, emotion regulation skills are related to improvement in symptoms following treatment of depressive, anxiety, substance use, eating, and borderline personality disorders (Sloan et al., 2017). Taken together, emotion dysregulation appears to be a key transdiagnostic construct relevant to many types of psychopathology across both internalizing and externalizing disorders (Carver et al., 2017).

Screening for emotion dysregulation is an important first step in understanding the role that emotion dysregulation plays in the development, maintenance, and treatment of psychopathology and other relevant psychological constructs (e.g., substance use, interpersonal conflict). Therefore, assessing emotion dysregulation is beneficial for many types of research studies (e.g., cross-sectional, longitudinal, and intensive longitudinal designs), clinical screening/intake procedures, and clinical outcome tracking. Thus, we sought to develop a brief measure of emotion dysregulation to be used as a screening tool across research and clinical settings.

Several existing emotion regulation and dysregulation measures are often used in research and clinical settings, but they may not be ideal for screening purposes due to length, lack of breadth, or specificity to a particular clinical population. The Emotion Regulation Questionnaire (ERQ; Gross & John, 2003), Difficulties in Emotion Regulation Scale (DERS; Gratz & Roemer, 2004), and the affective instability subscale of the Personality Assessment Inventory–Borderline Features Scale (PAI-BOR; Morey, 1991) are three such measures. The ERQ (Gross & John, 2003), for instance, focuses only on one aspect of Carpenter and Trull’s (2013) model of emotion dysregulation: the combination of inadequate appropriate regulation strategies and maladaptive regulation strategies. Specifically, the ERQ assesses the use of two emotion regulation strategies: one adaptive strategy (cognitive reappraisal) and one maladaptive strategy (expressive suppression), but it does not capture experiences central to emotion dysregulation more broadly (e.g., labile emotions, consequences of emotion dysregulation).

The DERS (Gratz & Roemer, 2004) uses an acceptance-based perspective to understand trait-level emotion regulation strategies and difficulties. The original DERS has a total of 36 items across six empirically derived subscales that focus on various emotion regulation problems (e.g., difficulties engaging in goal-directed behavior, lack of emotional clarity, impulse control difficulties). The DERS focuses heavily on outcomes and behaviors in the context of feeling upset. Thus, it captures consequences of emotion regulation, but not its core features, the experience of frequent severe and unstable negative emotions. In addition, with 36 items, the DERS is relatively lengthy and may not be an efficient screening instrument. More recently, three brief versions of the DERS have been developed (DERS-16, Bjureberg et al., 2016; DERS-18, Victor & Klonsky, 2016; DERS-SF, Kaufman et al., 2016), which range from 16 to 18 items. Two of the shortened measures (DERS-18, DERS-SF) retain the six-subscale structure, whereas the DERS-16 calculates a total score. Shortening the DERS remediated length concerns, but there currently is no consensus regarding which version best captures emotion regulation (Skutch et al., 2019). Furthermore, the items were taken from the original 36-item DERS, so the brief forms are affected by the same issues as their parent instrument such that they do not assess the experience of intense and unstable emotions.

The PAI-BOR (Morey, 1991) is a 24-item measure meant to assess features of BPD with four subscales. One six-item subscale is labeled affective instability (AI), which is often considered synonymous with emotion dysregulation. Although this subscale seeks to assess emotion dysregulation and is brief, an analysis of a large nonclinical sample failed to replicate the original factor structure of the PAI-BOR, with two items from the original six-item AI subscale loading more highly onto other factors (Jackson & Trull, 2001). For example, one of these two items was the reverse-coded item, “I am generally a happy person,” which may indicate a baseline mood state rather than a *fluctuation* of mood states. Although useful as an item meant to point to certain kinds of psychopathology (e.g., persistent depressive disorder), it might not be useful in assessing emotion *dysregulation*. Gardner and Qualter (2009) also failed to replicate the original factor structure of the AI subscale of the PAI-BOR in a nonclinical sample, finding a better fit from a four-factor solution in which four AI items (excluding the two regarding anger) were combined with items from other subscales to compose a factor of affective and self-dysregulation. Taken together, although the PAI-BOR AI subscale has been widely used in studies of BPD features (e.g., Baer & Sauer, 2011; Bagge et al., 2004; Trull et al., 1997), evidence suggests that it may not be appropriate for use as a measure of emotion dysregulation outside of a BPD context.

Limitations of existing emotion dysregulation measures highlight the need to develop a measure of emotion dysregulation outside of a theoretical context that is explicitly BPD-focused, given increased recognition that emotion dysregulation is a transdiagnostic construct. To address the issues described above, we developed the Brief Emotion Dysregulation Scale (BEDS). The BEDS increments existing measures in important ways. First, it intends to assess the broad construct of emotion dysregulation as opposed to narrower components of it. Specifically, the BEDS assesses aspects of emotion dysregulation that are missing from other measures: sensitivity to emotional stimuli, emotional lability, and consequences of emotion dysregulation (e.g., emotions causing interpersonal conflicts). Second, the BEDS assesses these components with a brief measure, which reduces burden and addresses length limitations of some of the measures described previously. Third, the BEDS evaluates emotion dysregulation outside of the context of BPD. All told, the development of the BEDS addresses challenges and limitations that may be present when clinicians and researchers seek to assess emotion dysregulation in time-limited contexts, such as for screening purposes. Finally, the BEDS is freely available, eliminating cost as a potential barrier to public use.

The current study had several aims: Aim 1a was to generate a large initial pool of items, Aim 1b was to assess content validity of the items, and Aim 1c was to explore and replicate the factor structure of the BEDS in independent samples. Aim 2 was to examine associations among the BEDS and existing emotion regulation measures and other theoretically relevant external criteria. Importantly, we examined the associations between BEDS subscales and measures of other forms of psychopathology to evaluate the validity of the BEDS with respect to its use as a transdiagnostic screening tool.

## Aim 1: Method and Results

### Aim 1a: Item Generation

Based on Carpenter and Trull (2013), we identified four domains of emotion dysregulation that we sought to measure: emotional sensitivity, emotional lability, emotional reactivity, and consequences of emotion dysregulation. We excluded emotion regulation strategies because those are thoroughly assessed by the DERS (Gratz & Roemer, 2004) and the ERQ (Gross & John, 2003), and because we were interested in developing a measure of the *experience* of emotion dysregulation and its consequences as opposed to strategies used during the experience of emotion dysregulation. The first, second, and last author (AMW, SAG, TJT, respectively) used working definitions of the four domains to create an initial set of 60 items with 15 per domain.

We defined **sensitivity** as *general negative emotionality; trait-like tendency, proneness, or temperament toward negative emotion; low threshold for emotions to occur*. We defined **lability** as *negative emotions changing frequently within daily life*. Finally, we defined **consequences** as *outcomes, internal (e.g., shame) or external (e.g., interpersonal conflict), resulting from emotion dysregulation*. Items were to be rated on a scale from 1 to 4 (1 = *strongly disagree*, 2 = *disagree*, 3 = *agree*, 4 = *strongly agree*). We defined **reactivity** as *heightened negative emotional response to environmental or internal stimuli or stress; rapid and strong emotional responses*. Although reactivity is not explicitly included in the Carpenter and Trull (2013) conceptualization of emotion dysregulation, we included it because it might conceptualize *strength* of emotional responses in a way that may be applicable to both clinical and nonclinical populations. Note that our scales focus on negative emotionality, as opposed to negative and positive emotionality.

### Aim 1b: Content Validity

We assessed the content validity of our initial pool of 60 items by providing our working definitions of the four domains to the third author (ACH) and three additional individuals with expertise in emotion dysregulation and then asking them to categorize each of the 60 items into which domains the items belonged. We selected the best 10 items from each domain based on number of correct classifications, resulting in a new set of 40 total items (see Table S1 for item content and descriptive statistics).

### Aim 1c: Factor Structure

#### Participants and Procedures

Study procedures were approved by the University of Missouri Institutional Review Board. All questionnaires were administered electronically.

##### Samples 1 and 2

Undergraduate students (*n* = 1,104) in introductory psychology courses completed the 40-item measure as part of a larger battery of self-report questionnaires to receive research credits at the beginning of the fall semester of 2020. These data were randomly divided into two samples (Sample 1 and Sample 2) that did not significantly differ on age, gender, or whether they currently were in treatment. Each of these sub-samples consisted of 552 participants. See Table 1 for demographic information by sample.

**Table 1.** Demographic and Sample Information.

Sample information	Sample 1	Sample 2	Sample 3	Sample 4	Sample 5
*N*	552	552	248	133	213
Age					
*M* (*SD*)	18.57 (1.74)	18.59 (1.59)	20.06 (3.45)	18.92 (1.28)	18.65 (1.71)
Range	17–51	17–39	18–66	18–24	17–39
Gender identity					
% Female	66	62	78	56	34
% Male	33	36	22	44	66
% Other	1	1	<1	0	0
Race/ethnicity					
% White	77.20	78.80	76.21	77.44	88.73
% Black	6.16	6.70	8.47	9.02	1.40
% Multiracial	10.50	8.15	8.47	6.02	5.63
% Other	6.16	6.34	6.85	5.26	4.22
% Currently in treatment	18.30	17.40	23.39	23.31	15.50
Sample purpose	Factor structure	Factor structure	Factor structure; validation	Validation	Validation

*Note.* Race/ethnicity was “check all that apply.” Sample 5 was a subset of Samples 1 and 2.

##### Sample 3

After factor analyses of the original 40 items, undergraduate students (*n* = 248) in an upper-level psychology course completed the final 12-item scale plus validation measures to receive extra credit in their course during fall semester of 2020. See Table 1 for demographic information by sample.

#### Exploratory Analyses

##### Analytic Method

Using data from Sample 1, we evaluated items iteratively based on endorsement rates, inter-item correlations within and across domains, alphas if-item-deleted within domains, corrected item-total correlations, and a series of exploratory factor analyses (EFAs). EFAs were conducted in Mplus version 7.4 using weighted least square mean and variance adjusted (WLSMV) estimation and geomin (an oblique) rotation. To determine number of factors and which items to retain, we examined eigenvalues, cross-loadings, and theoretical fit with the four domains of emotion dysregulation that we sought to measure. Finally, we considered root mean square error of approximation (RMSEA), comparative fit index (CFI), Tucker–Lewis index (TLI), and standardized root mean square residual (SRMR) when determining model fit.

##### Results

Reactivity items demonstrated high cross-loadings on our sensitivity and lability factors, suggesting that reactivity does not constitute a separable component of emotion dysregulation. In fact, reactivity might reflect the conjunction of sensitivity and lability: for instance, being reactive requires sensitivity to perceived interpersonal slights, and a sufficient degree of lability is necessary to constitute “reacting” to such a slight. Thus, we dropped the reactivity items from the scale. In addition, based on evidence from the EFAs, we determined that the consequences items would be more appropriate as a separate “problems” scale rather than its own factor within the larger scale. The consequences items did not form their own factor in the EFA. Rather, consequences items spread across the remaining factors. Thus, we elected to treat consequences as a separate scale to avoid conflating core emotion dysregulation features and consequences of dysregulation. Such an approach is compatible with broader movements to disentangle basic tendencies and dysfunction associated with basic tendencies, given that mechanisms implicated in dysfunction may be etiologically distinct from core features causing emotion dysregulation.

EFAs for the remaining items resulted in a six-item, two-factor solution for sensitivity and lability. The accompanying consequences scale was reduced from 10 to 6 items based on endorsement rates, inter-item correlations within domain, and item correlations with the sensitivity and lability subscales. As such, the BEDS comprises 12 items in total, with three items for sensitivity, three items for lability, and six items for consequences. Item content and descriptive statistics for the final scale are reported in Table 2, and factor loadings and model fit indices for the final EFA are presented in Table 3. The two-factor model, with correlated sensitivity and lability factors (*r* = .44), provided an excellent fit to the data (RMSEA = .018, CFI = 1.00, TLI = .999, SRMR = .009), and factors’ reliability was strong (alphas and omegas >.76).

**Table 2.** BEDS Item Content and Descriptive Information.

Subscale/item	Sample 1 (*N* = 552)	Sample 2 (*N* = 552)	Sample 3 (*N* = 248)	Sample 4 (*N* = 133)	Sample 5 (*N* = 213)
	*M* (*SD*)	*M* (*SD*)	*M* (*SD*)	*M* (*SD*)	*M* (*SD*)
Sensitivity (range = 1–4)	2.41 (0.77)	2.31 (0.75)	2.41 (0.71)	2.18 (0.71)	2.29 (0.77)
S3. I have a thick skin. [R]	2.34 (0.87)	2.19 (0.87)	2.37 (0.86)	2.18 (0.83)	2.18 (0.90)
S4. I cry easily.	2.30 (0.98)	2.22 (0.96)	2.35 (0.89)	2.06 (0.93)	2.17 (0.97)
S8. I’ve always been a sensitive person.	2.59 (0.94)	2.53 (0.91)	2.50 (0.86)	2.31 (0.93)	2.53 (0.94)
Lability (range = 1–4)	2.26 (0.70)	2.22 (0.66)	2.13 (0.61)	2.23 (0.65)	2.30 (0.70)
L3. It’s hard to predict my emotions from one moment to the next.	2.13 (0.83)	2.12 (0.79)	1.93 (0.75)	2.08 (0.81)	2.22 (0.85)
L6. I can feel one way in one minute and feel totally different in the next.	2.31 (0.87)	2.26 (0.87)	2.19 (0.83)	2.24 (0.86)	2.32 (0.85)
L9. My emotions about people or events change frequently.	2.34 (0.78)	2.30 (0.77)	2.28 (0.68)	2.37 (0.75)	2.35 (0.76)
Consequences					
Original scoring					
Consequences average (range = 1–4)	2.24 (0.60)	2.19 (0.55)	2.30 (0.56)	2.31 (0.56)	2.24 (0.57)
C1. My emotions cause problems or conflicts with other people.	2.14 (0.81)	2.08 (0.79)	2.16 (0.83)	2.18 (0.78)	2.13 (0.79)
C2. When I’m emotional, I don’t make the best decisions.	2.41 (0.81)	2.41 (076)	2.50 (0.73)	2.50 (0.72)	2.46 (0.80)
C3. When I’m emotional, I do things that I later regret.	2.10 (0.82)	2.09 (0.79)	2.19 (0.78)	2.16 (0.81)	2.14 (0.77)
C6. It bothers me that I’m so emotional.	2.21 (0.91)	2.09 (0.86)	2.17 (0.84)	2.14 (0.89)	2.15 (0.87)
C7. My emotions rarely cause problems for me. [R]	2.29 (0.82)	2.22 (0.77)	2.42 (0.81)	2.46 (0.86)	2.27 (0.80)
C8. My emotions don’t get the best of me. [R]	2.29 (0.79)	2.43 (0.77)	2.39 (0.72)	2.41 (0.76)	2.30 (0.76)
Dichotomized scoring					
Consequences count (range = 0–6)	2.13 (1.88)	1.93 (1.82)	2.46 (1.96)	2.38 (1.98)	2.16 (1.90)
C1. My emotions cause problems or conflicts with other people.	0.30 (0.46)	0.28 (0.45)	0.35 (0.48)	0.32 (0.47)	0.32 (0.47)
C2. When I’m emotional, I don’t make the best decisions.	0.45 (0.50)	0.45 (0.50)	0.50 (0.50)	0.49 (0.50)	0.50 (0.50)
C3. When I’m emotional, I do things that I later regret.	0.28 (0.45)	0.27 (0.44)	0.34 (0.48)	0.34 (0.47)	0.30 (0.46)
C6. It bothers me that I’m so emotional.	0.33 (0.47)	0.28 (0.45)	0.35 (0.48)	0.33 (0.47)	0.32 (0.47)
C7. My emotions rarely cause problems for me. [R]	0.38 (0.49)	0.33 (0.47)	0.49 (0.50)	0.44 (0.50)	0.36 (0.48)
C8. My emotions don’t get the best of me. [R]	0.37 (0.48)	0.34 (0.47)	0.43 (0.50)	0.46 (0.50)	0.38 (0.49)

*Note.* BEDS = Brief Emotion Dysregulation Scale; [R] = reverse-scored item.

**Table 3.** Item Loadings, Factor Correlations, Model Fit, Reliability, and Factorial Invariance for Final Two-Factor EFA and CFAs.

Factor analytic results	Sample 1 EFA		Sample 2 CFA		Sample 3 CFA	
*N*	552		552		248	
Standardized loadings (*SE*)						
Sensitivity	F1	F2				
S3	.75 (.04)	−.04 (.05)	.68 (.03)		.65 (.05)	
S4	.78 (.04)	.04 (.05)	.86 (.03)		.84 (.04)	
S8	.77 (.03)	.00 (.00)	.75 (.03)		.75 (.04)	
Lability						
L3	.05 (.05)	.70 (.04)	.68 (.03)		.73 (.06)	
L6	-.02 (.01)	.97 (.02)	.83 (.03)		.82 (.05)	
L9	.17 (.05)	.66 (.04)	.75 (.03)		.68 (.04)	
Factor correlation (*SE*)	.44 (.05)		.58 (.04)		.56 (.06)	
Model fit						
RMSEA	.018		.057		.048	
CFI	1.00		.992		.994	
TLI	.999		.986		.989	
SRMR	.009					
WRMR			.591		.465	
Factor reliability	Sensitivity	Lability	Sensitivity	Lability	Sensitivity	Lability
Alpha	.76	.80	.75	.75	.74	.72
Omega	.76	.81	.75	.75	.75	.73
Factorial invariance for gender						
Configural invariance						
RMSEA			.076			
CFI			.986			
TLI			.974			
WRMR			.800			
Metric invariance						
RMSEA			.081			
CFI			.981			
TLI			.971			
WRMR			1.008			
Scalar invariance						
RMSEA			.084			
CFI			.968			
TLI			.968			
WRMR			1.355			

*Note.* EFA = exploratory factor analysis; CFA = confirmatory factor analysis; RMSEA = root mean square error of approximation; CFI = comparative fit index; TLI = Tucker–Lewis index; SRMR = standardized root mean square residual; WRMR = weighted root mean square residual.

#### Confirmatory Analyses

##### Analytic Method

Using data from Sample 2, we conducted a confirmatory factor analysis (CFA) in Mplus using WLSMV estimation to confirm the two-factor structure for sensitivity and lability from our EFA. Loadings for the first item in each factor were set to 1.0. We also conducted a multigroup CFA to examine factorial invariance across gender in Sample 2. Specifically, we examined configural, metric, and strong invariance following procedures by Meredith (1993). Finally, we conducted another CFA in Sample 3. We examined RMSEA, CFI, and TLI to assess model fit.

##### Results

Item descriptive statistics for Samples 2 and 3 are reported in Table 2, and standardized factor loadings and model fit indices for CFAs in Samples 2 and 3 are reported in Table 3. The two-factor solution provided an excellent fit (Sample 2: RMSEA = .057, CFI = .992, TLI = .986; Sample 3: RMSEA = .048, CFI = .994, TLI = .989) and the factors were moderately correlated (Sample 2 *r* = .58, Sample 3 *r* = .56). Factors’ reliability was also strong for both samples (alphas and omegas >.72).

For the subsample of individuals from Sample 2 who reported their gender identity as male or female (*N* = 545; 344 female), the two-factor multigroup CFA showed strong factorial invariance for gender (RMSEA = .084, CFI = .968, TLI = .968). See Table 3 for fit across levels of invariance.

### Aim 2: Method and Results

#### Participants and Procedures

Study procedures were approved by our University Institutional Review Board. All questionnaires were administered electronically.

##### Sample 3

Undergraduate students (*n* = 248) in an upper-level psychology course completed the final scale and validation measures to receive extra credit points in their course. This sample was also used in a CFA for Aim 1c.

##### Sample 4

Undergraduate students (*n* = 133) in introductory psychology courses who did not complete the initial self-report battery (i.e., were not included in Samples 1 and 2) completed the final scale and validation measures. These students received course research credits for their participation. See Table 1 for demographic information.

##### Sample 5

A subset of 213 individuals from Samples 1 and 2 completed additional measures of psychopathology to receive course research credits, after completing the initial large battery of self-report questionnaires. Demographic details are listed in Table 1.

### Measures

#### BEDS

Based on results from Aim 1, the final BEDS comprises 12 items that are rated on a scale of 1 to 4 (1 = *strongly disagree*, 2 = *disagree*, 3 = *agree*, 4 = *strongly agree*). The BEDS includes a sensitivity subscale (3 items), a lability subscale (3 items), and an accompanying consequences subscale as an index of severity or impairment associated with emotion dysregulation (6 items). To examine the construct validity of the BEDS and its subscales, we aggregated the BEDS as follows. First, we created sensitivity and lability subscales by averaging the three items within each domain. Given that the CFA solutions revealed relatively tau equivalent factors, unit weighting is probably defensible and is most consistent with how screening scales are likely to be used in clinical settings. Second, we averaged the consequences items to create a consequences subscale. Third, to facilitate use in clinical settings, we dichotomized the consequences items such that ratings of 3 (*agree*) or 4 (*strongly agree*) were coded as 1, else 0, and then summed the dichotomized items to create a count score of number of consequences endorsed. Table 2 presents descriptive statistics for the BEDS in Samples 4 and 5. For our recommended administration and scoring procedures, see the Supplemental Material.

#### Existing Emotion Regulation Measures (Samples 3 and 4)

Supplemental Table S2 presents descriptive information for existing emotion regulation measures in Samples 3 and 4.

##### Difficulties With Emotion Regulation Scale

The DERS (Gratz & Roemer, 2004) is a 36-item measure of emotion regulation strategies and difficulties employing those strategies. Each item was rated on a scale of 1 (*almost never*) to 5 (*almost always*), and responses were summed to create a total score as well as six subscales (nonacceptance of emotional responses, difficulty engaging in goal-directed behavior, impulse control difficulties, lack of emotional awareness, limited access to emotion regulation strategies, lack of emotional clarity). As the three shorter versions of the DERS use items taken from the original DERS, we also computed total sum scores for the DERS-18 (18 items; Victor & Klonsky, 2016), DERS-SF (18 items; Kaufman et al., 2016), and DERS-16 (16 items; Bjureberg et al., 2016). Sums for each DERS scale were computed for participants with complete data on that particular scale. Higher scores indicate greater difficulty in regulation of emotions.

##### Emotion Regulation Questionnaire

The ERQ (Gross & John, 2003) is a 10-item measure of two emotion regulation strategies, cognitive reappraisal and expressive suppression, each with its own subscale. Items were rated on a scale from 1 (*strongly disagree*) to 7 (*strongly agree*), and responses were summed to create two total scores, one for each subscale, for participants with complete data on that particular subscale. Cognitive reappraisal comprises six items, and expressive suppression comprises four items. Higher scores on cognitive reappraisal indicate greater use of this adaptive emotion regulation strategy, while higher scores on expressive suppression indicate greater use of this maladaptive emotion regulation strategy.

##### Personality Assessment Inventory–Borderline Scale, Affective Instability Subscale

The PAI-BOR (Morey, 1991) is a 24-item measure of features of BPD. It contains four subscales with six items each: affective instability, identity disturbance, negative relationships, and self-harm. Each item was rated on a scale of 0 (*false, not true at all*) to 3 (*very true*), and responses were summed to create a total score and four subscale scores for participants with complete data on the particular subscale. We used the affective instability (AI) subscale as a primary convergent measure. Higher scores on this subscale indicate greater affective instability.

##### Perth Emotional Reactivity Scale

The PERS (Becerra et al., 2019) is a self-report measure of positive- and negative-emotional reactivity. For both positive and negative reactivity, there is a total score and three subscales (i.e., activation, intensity, and duration of emotional responses). The PERS contains 30 items, all rated on 5-point scale from 1 (*very unlike me*) to 5 (*very like me*). The present study focused on the negative reactivity domain, including the total score (general negative reactivity) and the three associated subscales. Scales were computed by summing respective items for participants with complete data on the particular subscale. Higher scores indicate more emotional reactivity.

#### External Validators (Samples 3 and 4)

Supplemental Table S3 presents descriptive information for measures of external validators in Samples 3 and 4.

##### PAI-BOR Total Score and Subscales

In addition to the PAI-BOR affective instability subscale (described earlier, *Primary Convergent Measures*), we used the PAI-BOR total score and three other subscales (i.e., identity, negative relationships, self-harm) in our examination of criterion validity. Higher scores indicate greater BPD features.

##### Urgency, Premeditation, Perseverance, Sensation Seeking, and Positive Urgency Impulsive Behavior Scale

The Urgency, Premeditation, Perseverance, Sensation Seeking, and Positive Urgency Impulsive Behavior Scale (UPPS-P; Lynam et al., 2006) is a 59-item measure of five dimensions of impulsivity: (Negative and Positive) Urgency, (lack of) Premeditation, (lack of) Perseverance, and Sensation Seeking. Each item was rated on a scale from 1 (*agree strongly*) to 4 (*disagree strongly*), and responses were averaged to create four subscale scores for participants with no more than one missing item in that particular subscale. Higher scores indicate greater impulsive traits. Because of mixed findings in the literature and the nonclinical nature of our sample, we did not expect Sensation Seeking to correlate with and scale in the BEDS.

##### Acceptance and Action Questionnaire-II

The Acceptance and Action Questionnaire-II (AAQ-II; Bond et al., 2011) is a seven-item measure of psychological inflexibility or experiential avoidance (e.g., “I’m afraid of my feelings.”). Each item was rated on a scale from 1 (*never true*) to 7 (*always true*), and responses were summed to create a total score for participants with complete data on the scale. Higher scores represent greater psychological inflexibility.

##### Alcohol Use Disorders Identification Test

The Alcohol Use Disorders Identification Test (AUDIT; Saunders et al., 1993) is a 10-item measure of alcohol consumption and related problems. Each item was rated on a scale from 0 to 4, where response options are generally worded to span a low to high frequency of occurrence. Responses were summed to create a total score for participants with complete data on the scale. Higher scores indicate greater alcohol use and related problems.

##### Drug Use Disorders Identification Test

The Drug Use Disorders Identification Test (DUDIT; Berman et al., 2005) is an 11-item measure of use of substances other than alcohol, and related problems. Each item was rated on a scale from 0 to 4, where response options are generally worded to span a low to high frequency of occurrence. Responses were summed to create a total score for participants with complete data on the scale. Higher scores indicate greater substance use and related problems.

##### Deliberate Self-Harm Inventory

The Deliberate Self-Harm Inventory (DSHI; Gratz, 2001) is a 17-item measure of frequency of engagement in various deliberate self-harming behaviors. We created three variables: (a) dichotomous variable indicating whether each participant had ever engaged in any deliberate self-harm, (b) self-reported number of lifetime instances engagement in self-harm, (b) continuous variable indicating the number of different methods reported by each participant.

##### Ruminative Response Scale

The Ruminative Response Scale (RRS; Treynor et al., 2003) is a 22-item measure of rumination as a response style to depression. Each item was rated on a scale from 1 (*almost never*) to 4 (*almost always*), and responses were summed to create a total score for participants with complete data on the scale. Higher scores indicate greater rumination.

##### Personality Inventory for *DSM*-5 Brief Form

The Personality Inventory for *DSM*-5 Brief Form (PID-5-BF; American Psychiatric Association [APA], 2013; Anderson et al., 2018) assesses traits of the *Diagnostic and Statistical Manual of Mental Disorders* (5th ed.; *DSM-5*; APA, 2013) alternative model of personality disorder (negative affectivity, detachment, antagonism, disinhibition, and psychoticism) with five subscales (five items each), all rated on a scale from 0 (*very false or often false*) to 3 (*very true or often true*). The items for each subscale were averaged for a subscale score when participants had responses for at least four of the five items. Higher scores indicate higher levels of the respective trait.

##### Five Facet Mindfulness Questionnaire

The Five Facet Mindfulness Questionnaire (FFMQ; Baer et al., 2006) is a 39-item self-report measure with a total score and five subscales (e.g., observing, describing). All items are rated on a 5-point scale ranging from 1 (*never or very rarely true*) to 5 (*very often or always true*). For the present study, we focused on the total score, which was calculated by summing all scale items for participants with complete data on the measure. Higher scores reflect higher levels of mindfulness.

##### Patient Health Questionnaire Depression Module

The Patient Health Questionnaire Depression Module (PHQ-9; Kroenke et al., 2001) is a self-report screening measure for major depressive disorder. All nine *DSM-5* depression symptoms for the past 2 weeks are assessed using a 4-point rating scale from 0 (*not at all*) to 3 (*nearly every day*). A total score was computed by summing all items for participants with complete data across the items. Higher scores reflect more severe depression symptoms.

##### Generalized Anxiety Disorder Scale

**The Generalized Anxiety Disorder Scale** (GAD-7; Spitzer et al., 2006) assesses past 2-week symptoms of generalized anxiety disorder. Participants rate each symptom on a 4-point scale from 0 (*not at all*) to 3 (*nearly every day*). A total score was computed by summing all items for participants with complete data across the items. Higher scores reflect more severe anxiety symptoms.

##### Treatment

Participants answered a single yes or no question regarding involvement in current psychiatric treatment: “Are you currently in treatment (i.e., taking medications or receiving therapy) for an emotional or psychological problem?”

##### Psychopathology Measures (Sample 5)

Supplemental Table S4 presents descriptive information for psychopathology measures in Sample 5.

###### Inventory of Depression and Anxiety Symptoms-II

The Inventory of Depression and Anxiety Symptoms-II (IDAS-II) is a 99-item measure that assesses various domains of psychopathology, including depressive symptoms, insomnia, panic, social anxiety, and others (Watson et al., 2012). Items were rated on a 5-point scale ranging from 1 (*not at all*) to 5 (*extremely*). For the present study, we focused on depressive symptomology (e.g., dysphoria, appetite loss, general wellbeing), using a general depression subscale that includes core affective and cognitive symptoms relevant to depression and anxiety (Watson et al., 2012).

###### Anxiety Sensitivity Index

The Anxiety Sensitivity Index (ASI) is a 16-item self-report measure of anxiety sensitivity, or the fear and worry associated with physiological symptoms of anxiety, such as increased heart rate or feeling shaky (Rodriguez et al., 2004). Each item is rated on a 5-point scale ranging from 1 (*very little*) to 5 (*very much*). A total score was computed by summing all items for participants with complete data on the measure. Higher scores reflect higher levels of sensitivity to the physiological symptoms of anxiety.

###### Externalizing Spectrum Inventory Brief Form

The Externalizing Spectrum Inventory Brief Form (ESI-BF) is a 160-item questionnaire that yields a total score, three higher order dimensions/factors (i.e., general disinhibition, callous-aggression, and substance abuse) and 23 subscales (Patrick et al., 2013). For the present study, we focused on the total score and three factor scores. A 4-point Likert-type scale included the following response options: true, somewhat true, somewhat false, and false.

### Analytic Method

Zero-order correlations for the BEDS with convergent measures were conducted using Samples 3 and 4. To compare the performance of the BEDS to that of similar existing scales, we also computed convergent correlations for the DERS (Gratz & Roemer, 2004), DERS short forms (Bjureberg et al., 2016; Kaufman et al., 2016; Victor & Klonsky, 2016), and PERS (Becerra et al., 2019). We also conducted a *t* test to determine whether the BEDS significantly differed based on whether participants reported being in treatment, as a preliminary examination of the clinical relevance of the BEDS. Again, other psychopathology data were available only in sample 5. Missing data were handled using pairwise deletion, and we applied Bonferroni corrections for *p* values within each sample.

### Results

#### Convergent Correlations With Existing Emotion Regulation Measures

Correlations between the BEDS subscales (sensitivity, lability, and both consequences scoring versions) and existing emotion regulation measures are presented in Table 4. To compare the performance of the BEDS to that of similar existing measures, correlations with primary convergent measures for the DERS total score, DERS short form total scores, and the PERS are presented in Supplemental Table S5. The BEDS subscales largely showed significant associations in expected directions with existing emotion regulation measures in both samples 3 and 4. In particular, the BEDS subscales showed moderate to large positive correlations with DERS total score and all three of the DERS short form total scores in both samples. The BEDS subscales also showed generally moderate to large positive correlations with the DERS subscales, PERS Negative total score and PERS Negative subscales, and the PAI-BOR affective instability scale, with the following exceptions. BEDS subscales were not significantly correlated with the awareness subscale of the DERS, BEDS sensitivity was not correlated with PAI-BOR affective instability or PERS intensity or duration in Sample 4, and the BEDS lability and consequences subscales were not robustly associated with ERQ subscales. Comparison with Table S5 demonstrates a similar pattern of correlations for the DERS total score, DERS short form total scores, and PERS negative total score with other emotion regulation measures.

**Table 4.** Zero-Order Correlations Among the BEDS and Existing Emotion Regulation Measures in Samples 3 and 4.

	BEDS scale			
Measure	Sensitivity	Lability	Consequences scale	Consequences count
DERS total score	**.38 /.38**	**.63 /.45**	**.61 /.62**	**.57 /.58**
DERS nonacceptance	**.29 /**.20	**.43 /.36**	**.42 /.47**	**.39 /.45**
DERS goals	**.38 /.41**	**.44 /**.29	**.51 /.50**	**.42 /.46**
DERS impulse	**.36 /.34**	**.61 /.40**	**.58 /.60**	**.57 /.54**
DERS awareness	−.04 **/**−.05	.21 **/**.17	.17 **/**.09	.19 **/**.11
DERS strategies	**.41 /.41**	**.63 /.38**	**.60 /.59**	**.57 /.54**
DERS clarity	**.25 /**.29	**.46 /.32**	**.43 /**.25	**.42 /**.24
DERS-18 total score	**.35 /.38**	**.61 /.45**	**.57 /.59**	**.54 /.56**
DERS-SF total score	**.35 /.40**	**.62 /.43**	**.57 /.60**	**.54 /.56**
DERS-16 total score	**.42 /.41**	**.63 /.42**	**.61 /.63**	**.57 /.58**
ERQ cognitive reappraisal	−.16 **/**−.03	−**.31 /**−.10	−**.31 /**−.25	−**.26 /**−.28
ERQ expressive suppression	−.20 **/**−.14	−.00 **/**−.01	.05 **/**−.02	.05 **/**−.04
PAI-BOR affective instability	**.45 /**.19	**.66 /.65**	**.63 /.57**	**.62 /.54**
PERS Negative total	**.40 /.34**	**.51 /.42**	**.55 /.54**	**.52 /.51**
Activation	**.40 /.34**	**.50 /.42**	**.54 /.53**	**.52 /.50**
Intensity	**.33 /**.30	**.45 /.39**	**.46 /.49**	**.44 /.46**
Duration	**.40 /**.31	**.49 /.38**	**.56 /.49**	**.52 /.47**

*Note.* Correlations significant at Bonferroni corrected *p* < .00030 are bolded. Missing data were handled using pairwise deletion, resulting in *n*s for the correlations in this table ranging from 233 to 248 for the Sample 3 and 127 to 131 for Sample 4. Correlations for Sample 3 appear to the left of the “/” and those for Sample 4 appear to the right. BEDS = Brief Emotion Dysregulation Scale; DERS = Difficulties in Emotion Regulation Scale (Gratz & Roemer, 2004); DERS-SF = difficulties in emotion regulation scale short form; ERQ = Emotion Regulation Questionnaire (Gross & John, 2003); PAI-BOR = Personality Assessment Inventory–Borderline Features Scale (Morey, 1991); PERS = Perth Emotional Reactivity Scale (Becerra et al., 2019).

#### Correlations With Theoretically Relevant External Criteria

Correlations between the BEDS subscales and other external validators are presented in Table 5. To compare the performance of the BEDS to that of similar existing measures, correlations with associated constructs for the DERS total score, DERS short form total scores, and the PERS are presented in Supplemental Table S6. The BEDS subscales largely showed significant associations in expected directions with associated constructs in both samples 3 and 4. Specifically, all BEDS subscales showed small to large positive correlations with PAI-BOR total score, all remaining PAI-BOR subscales other than affective instability (identity disturbance, negative relationships, and self-harm), UPPS-P negative urgency and (lack of) perseverance, AAQ-II, DSHI yes/no, RRS, PID-5-BF negative affectivity, PHQ-9, and GAD-7, in both samples. In addition, all BEDS subscales showed moderate to large negative correlations with the FFMQ in both samples. Furthermore, in both samples, BEDS lability and consequences subscales showed moderate positive correlations with UPPS-P positive urgency, DSHI number of methods, and PID-5-BF detachment, antagonism, disinhibition, and psychoticism. The BEDS lability scale showed small to moderate positive correlations with the AUDIT and DUDIT total scores in both samples, and the BEDS consequences count scoring showed small correlations with DSHI frequency in both samples. Finally, the BEDS sensitivity and consequences subscales showed small to moderate correlations with being in treatment in both samples. Comparison with Table S6 demonstrates a similar pattern of correlations for the DERS total score, DERS short form total scores, and PERS negative total score with associated constructs.

**Table 5.** Zero-Order Correlations Among the BEDS and External Validators in Samples 3 and 4.

	BEDS scale			
Measure	Sensitivity	Lability	Consequences scale	Consequences count
PAI-BOR total score	**.45 /.35**	**.64 /.57**	**.63 /.67**	**.62 /.61**
PAI-BOR identity disturbance	**.45 /.38**	**.55 /.41**	**.56 /.53**	**.56 /.49**
PAI-BOR negative relationships	**.26 /.37**	**.40 /.38**	**.39 /.61**	**.40 /.53**
PAI-BOR self-harm	**.24 /.10**	**.46 /.31**	**.41 /.33**	**.40 /.32**
UPPS-P negative urgency	**.29 /.17**	**.47 /.35**	**.56 /.56**	**.54 /.50**
UPPS-P (lack of) premeditation	.02 /−.01	.16 /.11	.11 /.09	.11 /.08
UPPS-P (lack of) perseverance	**.31 /.22**	**.38 /.15**	**.36 /.23**	**.35 /.24**
UPPS-P sensation seeking	**−.24 /−.32**	**−**.00 /**−**.05	**−**.09 /**−**.11	**−**.11 /**−**.13
UPPS-P positive urgency	.10 /.03	**.38 /.27**	**.37 /.35**	**.35 /.31**
AAQ-II	**.35 /.39**	**.54 /.37**	**.59 /.56**	**.58 /.51**
AUDIT	.01 /.09	**.28 /.14**	.20 /.08	.18 /.04
DUDIT	**−**.01 /.10	**.26 /.08**	.19 /.08	.20 /.01
DSHI yes/no	**.26 /.30**	**.35 /.12**	**.40 /.30**	**.38 /.24**
DSHI frequency	.11 /.16	.21 /**−**.05	.15 /.06	**.15 /.05**
DSHI number of methods	.21 /**.31**	**.35 /.11**	**.37 /.25**	**.37 /.21**
RRS	**.30 /.37**	**.50 /.35**	**.48 /.48**	**.46 /.43**
PID-5-BF negative affectivity	**.54 /.51**	**.50 /.43**	**.57 /.53**	**.55 /.51**
PID-5-BF detachment	.14 /.13	**.35 /.27**	**.32 /.31**	**.32 /.29**
PID-5-BF antagonism	.14 /.09	**.32 /.32**	**.24 /.34**	**.26 /.31**
PID-5-BF disinhibition	.11 /.04	**.37 /.27**	**.29 /.33**	**.31 /.28**
PID-5-BF psychoticism	.22 /.18	**.44 /.28**	**.36 /.35**	**.38 /.31**
FFMQ	**−.29 /−.24**	**−.48 /−.31**	**−.51 /−.38**	**−.49 /−.38**
PHQ-9	**.26 /.29**	**.42 /.26**	**.40 /.44**	**.39 /.44**
GAD-7	**.32 /.41**	**.44 /.36**	**.50 /.47**	**.47 /.46**
In treatment	**.24 /.14**	.31 /.05	**.28 /.15**	**.29 /.18**

*Note.* Correlations significant at Bonferroni corrected *p* < .00030 are bolded. Missing data were handled using pairwise deletion, resulting in *n*s for the correlations in this table ranging from 238 to 248 for Sample 3 and 127 to 132 for Sample 4. Correlations for Sample 3 appear to the left of the “/” and those for Sample 4 appear to the right. BEDS = Brief Emotion Dysregulation Scale; PAI-BOR = Personality Assessment Inventory–Borderline Features Scale (Morey, 1991); UPPS-P = Urgency, Premeditation, Perseverance, Sensation Seeking, and Positive Urgency Impulsive Behavior Scale (Lynam et al., 2006); AAQ-II = Acceptance and Action Questionnaire-II (Bond et al., 2011); AUDIT = Alcohol Use Disorders Identification Test (Saunders et al., 1993); DUDIT = Drug Use Disorders Identification Test (Berman et al., 2005); DSHI = Deliberate Self-Harm Inventory (Gratz, 2001); RRS = Ruminative Response Scale (Treynor et al., 2003); PID-5-BF = Personality Inventory for *DSM-5* Brief Form (Anderson et al., 2018); FFMQ = Five Facet Mindfulness Questionnaire (Baer et al., 2006); PHQ-9 = Patient Health Questionnaire Depression Module (Kroenke et al., 2001); GAD-7 = Generalized Anxiety Disorder scale (Spitzer et al., 2006).

#### Other Psychopathology Measures

Correlations between the BEDS subscales (sensitivity, lability, and both consequences scoring versions) and psychopathology measures in Sample 5 are presented in Table 6. The BEDS subscales showed moderate to large positive correlations with IDAS-II general depression and ASI total score. The lability and consequences subscales of the BEDS also showed moderate to large positive correlations with ESI general disinhibition. The BEDS subscales were not significantly correlated with ESI total, callous-aggression, or substance abuse scores.

**Table 6.** Zero-Order Correlations Among the BEDS and Psychopathology Measures in Sample 5.

	BEDS scale			
Measure	Sensitivity	Lability	Consequences scale	Consequences count
IDAS-II general depression	**.25**	**.42**	**.43**	**.39**
ASI total score	**.31**	**.32**	**.30**	**.25**
ESI total score	.04	.18	.22	.22
ESI general disinhibition	.19	**.27**	**.36**	**.38**
ESI callous-aggression	−.13	.05	.04	.07
ESI substance abuse	−.01	.10	.08	.05

*Note.* Correlations significant at Bonferroni corrected *p* < .00208 are bolded. Missing data were handled using pairwise deletion, resulting in *n*s ranging from 181 to 210 for the correlations in this table. BEDS = Brief Emotion Dysregulation Scale; IDAS-II = Inventory of Depression and Anxiety Symptoms-II (Watson et al., 2012); ASI = Anxiety Sensitivity Index (Rodriguez et al., 2004); ESI = Externalizing Symptom Inventory Brief Form (Patrick et al., 2013).

## Discussion

The current study sought to develop and examine the construct validity of a Brief Emotion Dysregulation Scale (BEDS) for use as a brief, transdiagnostic screening tool in research and clinical settings. Existing measures of emotion dysregulation (or regulation) largely assess use of specific emotion regulation strategies (e.g., DERS, Gratz & Roemer, 2004; ERQ, Gross & John, 2003) or affective instability within the context of BPD (e.g., the PAI-BOR scale; Morey, 1991). As emotion dysregulation has been more recently conceptualized as a transdiagnostic construct (Sloan et al., 2017), a brief screening measure for emotion dysregulation in general is needed.

Based on the Carpenter and Trull (2013) model of emotion dysregulation, we generated a large pool of initial items covering four components: emotional sensitivity, emotional reactivity, emotional lability, and consequences of emotion dysregulation. We elected not to assess emotion regulation strategies because those are thoroughly examined by other measures (e.g., DERS, Gratz & Roemer, 2004; ERQ, Gross & John, 2003) and because our focus for the BEDS was on screening as a first step, after which more thorough assessment of specific emotion regulation strategies might be indicated. Next, we retained a subset of the initial pool of items based on content validity. Finally, we iteratively evaluated the remaining items and explored the factor structure of the BEDS, resulting in a final scale that has a two-factor solution including subscales for sensitivity and lability, along with an accompanying consequences subscale to index problems associated with emotion dysregulation. With three items for sensitivity and lability each, along with six consequences items, the BEDS includes 12 items total, supporting its use as a brief screening tool. We replicated the two-factor solution for sensitivity and lability in two independent samples and demonstrated factorial invariance across levels of gender.

A combination of empirical evidence and conceptual support led us to drop the reactivity items from the final scale. EFA results indicated that the reactivity items did not form their own factor. Instead, reactivity items loaded highly on the sensitivity and lability domains. Consistent with this observation, there is relatively weaker theoretical rationale for reactivity as its own domain, compared with sensitivity and lability. The model of emotion dysregulation on which we based our scale does not specify emotional reactivity as a separate component of the process of emotion dysregulation (Carpenter & Trull, 2013). It seems plausible that reactivity is a redundant component of emotion dysregulation after sensitivity and lability are represented. In other words, reactivity may reflect the combination of both sensitivity and lability. In creating our scale items, we conceptualized emotional reactivity as a dimensional representation of the strength of negative emotions, which may or may not be directly related to emotion dysregulation (e.g., a person may report experiencing negative emotions more acutely when they occur with or without reporting increased lability). While the reactivity domain may be a useful supplement to understanding emotional functioning broadly, these results suggest a less specific role of reactivity in emotion dysregulation.

The pattern of correlations observed in Samples 3 and 4 between the BEDS and convergent measures supports the use of the BEDS to screen for emotion dysregulation. Specifically, with very few exceptions, the BEDS subscales were positively associated with emotion regulation difficulties, affective instability from a BPD context, and emotional reactivity. Furthermore, the BEDS lability and consequences scales were negatively associated with adaptive emotion regulation.

Adding to this, the pattern of correlations observed in Samples 3 and 4 between the BEDS and external validators (including psychopathology symptoms in Sample 5) further supports the construct validity of the BEDS and, notably, supports its use as a *transdiagnostic* screening tool. With few exceptions, the BEDS subscales in Samples 3, 4, and 5 were associated in the expected directions with features of borderline personality disorder, aspects of impulsivity, psychological inflexibility, self-harm, rumination, personality disorder traits, mindfulness, depressive symptoms, anxiety symptoms, and being in treatment for an emotional or psychological problem. In addition, BEDS lability was positively associated with symptoms of alcohol and other substance use disorders. In contrast, correlations among the various DERS forms, the PERS negative total score, and these associated constructs were only significant in one sample (Sample 3), indicating that the BEDS may perform better than existing scales in capturing emotion dysregulation and its relations with other psychological symptoms and distress.

Collectively, results from the present study indicate that the BEDS is an appropriate measure of two specific components of emotion dysregulation, sensitivity and lability, as well as consequences of emotion dysregulation. The BEDS shows good convergent validity in separate college student samples and performs as well or better than existing measures of emotion regulation and reactivity in terms of its associations with related psychological constructs. Furthermore, the BEDS is associated with a broad range of psychiatric symptoms spanning internalizing and externalizing domains, supporting its use as a transdiagnostic screening tool. Given its relatively short length, well supported factor structure, and good preliminary construct validity, the BEDS may be a useful tool in both research and clinical settings for the assessment of emotion dysregulation and associated impairment.

### Utility in Research Settings

By dimensionally capturing two components of emotion dysregulation and related consequences, the BEDS is well-suited for use as a transdiagnostic screening measure for research participant selection. The BEDS could be used by researchers interested in recruiting participants with emotion dysregulation based on sensitivity, lability, and the presence (or absence) of consequences rather than by specific diagnostic category. Furthermore, by assessing sensitivity and lability separately, the BEDS allows for examination of how sensitivity and lability may be differentially related to or exacerbate other co-occurring psychiatric problems. For example, sensitivity but not lability was positively associated with being in treatment and negatively associated with sensation seeking. In contrast, lability was positively associated with psychoticism and higher scores on measures of substance use, but sensitivity was not significantly associated with either construct. Moreover, given relatively low participant burden associated with responding to this measure, the BEDS could be administered multiple times over a study period, and future research could examine how separate dimensions of emotion regulation may change over time or across contexts using this freely available measure. Relatedly, future work might evaluate the appropriateness of using the BEDS in ecological momentary assessment, as its brevity lends itself well to intensive longitudinal data collection.

### Utility in Clinical Settings

The BEDS may be a helpful screening tool used at entry to treatment or during clinical intake procedures. Evaluating baseline emotion dysregulation regardless of diagnoses can provide key information for treatment given the transdiagnostic nature of emotion dysregulation. In addition, BEDS scores may indicate certain areas for further assessment. The BEDS may also be helpful in regular assessment and monitoring throughout treatment. Given the brief nature of the instrument, it can be easily administered at weekly, monthly, or other determined intervals to track progress and client experiences over the course of treatment. The distinction between consequences and other BEDS subscales (i.e., sensitivity, lability) may be particularly useful in treatment as the clinician and client can work together to track decreases in consequences, which would likely be clinically meaningful even if baseline emotional sensitivity, for example, did not decrease substantially. Relatedly, given that the sensitivity subscale showed a similar but generally weaker pattern of correlations with external validators and psychopathology symptoms compared with lability and consequences, clinicians may elect to focus on lability and consequences rather than sensitivity in treatment. That said, sensitivity is one of the components of the conceptual model of emotion dysregulation (Carpenter & Trull, 2013) and it was significantly correlated with being in treatment whereas lability was not, suggesting that the sensitivity subscale may be particularly relevant among individuals in treatment. Future work should further investigate the BEDS subscales with clinical utility and brevity in mind.

### Limitations and Future Directions

These studies had several limitations. Our samples included predominantly white college students, making it unclear how generalizable the current results will be to different age ranges, racial or ethnic identities, or clinical samples. Further examination is needed to inform appropriate use of the BEDS with different populations. Notably, we collected all data during the fall of 2020, during the Covid-19 pandemic. Given evidence that small changes to personality traits may have occurred over the course of the pandemic especially among emerging adults (e.g., Sutin et al., 2022), future work should seek to replicate the current factor structure of the BEDS. Another valuable next step for future work would be to assess the BEDS in community as well as clinical samples to develop normative data and identify cutoff scores that might indicate clinically relevant emotion dysregulation. This may enhance the utility of the BEDS in clinical settings and facilitate decision-making in treatment. In addition, this work should seek to replicate the present factor structure among clinical and community samples. Although our empirical reasons for dropping the reactivity subscale are accompanied by a theoretical rationale (Carpenter & Trull, 2013), our final scale does deviate from our hypothesized factor structure and replicating the resulting structure in clinical and community samples would support the use of the BEDS in such samples.

The associated constructs selected for validation of the BEDS were not comprehensive and do not cover all behaviors or clinical constructs of interest. Future work might examine additional indicators such as diagnostic status or more thorough assessment of treatment utilization to further clarify the scope of the BEDS. Finally, we acknowledge that, in its brevity, the BEDS cannot capture every aspect of emotion dysregulation to the extent that lengthier and more focused scales do (e.g., use of adaptive and maladaptive emotion regulation strategies). This limits the utility of the BEDS for comprehensive assessment purposes; however, we intend for the BEDS to complement existing measures by functioning as a brief, transdiagnostic screening tool that can serve as the first step in assessing emotion dysregulation in contexts where time and other resources are limited, after which more thorough assessment of specific emotion regulation strategies might be indicated.

## Conclusion

We developed a Brief Emotion Dysregulation Scale (BEDS) that assesses emotional sensitivity and lability as well as consequences of emotion dysregulation. A two-factor model for sensitivity and lability fit the data well and was invariant across levels of gender. Correlations with convergent measures, including psychiatric symptoms, supported the construct validity of the BEDS and its use as a transdiagnostic screening tool. Given its brevity, the BEDS may be a useful screening tool for research studies on emotion dysregulation and in clinical settings in which time and resources are limited. Further research should examine the validity and application of the BEDS as a screening measure in clinical samples.

## Supplemental Material

sj-docx-1-asm-10.1177_10731911231161800 – Supplemental material for The Brief Emotion Dysregulation Scale: Development, Preliminary Validation, and Recommendations for Use Supplemental material, sj-docx-1-asm-10.1177_10731911231161800 for The Brief Emotion Dysregulation Scale: Development, Preliminary Validation, and Recommendations for Use by Andrea M. Wycoff, Sarah A. Griffin, Ashley C. Helle, Alison M. Haney, Ashley L. Watts and Timothy J. Trull in Assessment
