# Supplementary material for: The Brief Emotion Dysregulation Scale: Development, Preliminary Validation, and Recommendations for Use
Source: Assessment. 2023 Mar 24;31(2):335–49. doi: 10.1177/10731911231161800 (PMC10518026; doi:10.1177/10731911231161800)
Supplement: sj-docx-1-asm-10.1177_10731911231161800 – Supplemental material for The Brief Emotion Dysregulation Scale: Development, Preliminary Validation, and Recommendations for Use [file sj-docx-1-asm-10.1177_10731911231161800.docx]

**BEDS**

Below are a series of statements that describe how people experience their emotions. For each statement, please rate how much you agree or disagree with the statement. If you strongly disagree with the statement, select 1. If you disagree with the statement, select 2. If you agree with the statement, select 3. If you strongly agree with the statement, select 4. Be sure to rate your agreement or disagreement for each of the statements below.

|  |  | **Strongly**  **disagree** | **Disagree** | **Agree** | **Strongly**  **agree** |
| --- | --- | --- | --- | --- | --- |
| 1. | I cry easily. | 1 | 2 | 3 | 4 |
| 2. | My emotions about people or events change frequently. | 1 | 2 | 3 | 4 |
| 3. | I've always been a sensitive person. | 1 | 2 | 3 | 4 |
| 4. | It's hard to predict my emotions from one moment to the next. | 1 | 2 | 3 | 4 |
| 5. | I can feel one way in one minute and feel totally different in the next. | 1 | 2 | 3 | 4 |
| 6. | I have a thick skin. | 1 | 2 | 3 | 4 |
| 7. | My emotions cause problems or conflicts with other people. | 1 | 2 | 3 | 4 |
| 8. | When I'm emotional, I don't make the best decisions. | 1 | 2 | 3 | 4 |
| 9. | When I'm emotional, I do things that I later regret. | 1 | 2 | 3 | 4 |
| 10. | It bothers me that I'm so emotional. | 1 | 2 | 3 | 4 |
| 11. | My emotions rarely cause problems for me. | 1 | 2 | 3 | 4 |
| 12. | My emotions don't get the best of me. | 1 | 2 | 3 | 4 |

**Scoring Instructions**

This measure uses 1 (strongly disagree) to 4 (strongly agree) response options so that higher scores indicate greater difficulties with the respective construct. **Because the scales include reverse-keyed items, it is important to reverse the scores of these items prior to calculating scale scores*.** For each scale, we recommend calculating the mean of the item scores. If you are interested in the variety of consequences related to emotion dysregulation, the consequences scale can be scored as a count score. To obtain the consequences count score, count each item rated “agree” or “strongly agree” (after reverse scoring), for a possible range of 0-6 consequences endorsed.

Sensitivity: 1, 3, 6 [R]

Lability: 2, 4, 5

Consequences: Items 7, 8, 9, 10, 11 [R], 12 [R]

*[R] indicates these items should be reverse-scored such that 1=4, 2=3, 3=2, and 4=1.

| **Table S1** |  |  |  |
| --- | --- | --- | --- |
| *BEDS original 40 item content and descriptive information* | | | |
|  | Sample 1 (*N*=552) |  | Sample 2 (*N*=552) |
| Subscale/Item | *M (SD)* |  | *M (SD)* |
| **Sensitivity** (range = 1 to 4) | 2.45 (0.63) |  | 2.38 (0.59) |
| S1. I rarely feel emotional. [R] | 3.00 (0.82) |  | 2.95 (0.83) |
| S2. I'm often sad or anxious. | 2.45 (0.90) |  | 2.43 (0.90) |
| S3. I have a thick skin. [R] | 2.34 (0.87) |  | 2.19 (0.87) |
| S4. I cry easily. | 2.30 (0.98) |  | 2.22 (0.96) |
| S5. I get emotionally hurt pretty easily. | 2.44 (0.90) |  | 2.38 (0.89) |
| S6. I'm more emotional than most people I know. | 2.37 (0.99) |  | 2.30 (0.84) |
| S7. I feel emotions very deeply. | 2.89 (0.85) |  | 2.79 (0.85) |
| S8. I've always been a sensitive person. | 2.59 (0.94) |  | 2.53 (0.91) |
| S9. I'm usually a happy or content person. [R] | 1.93 (0.66) |  | 1.95 (0.70) |
| S10. People tell me I'm an emotional person. | 2.16 (0.95) |  | 2.06 (0.93) |
| **Lability** (range = 1 to 4) | 2.33 (0.62) |  | 2.29 (0.60) |
| L1. My emotions can change a lot within a day. | 2.81 (0.81) |  | 2.80 (0.81) |
| L2. My emotions are very steady. [R] | 2.45 (0.70) |  | 2.43 (0.73) |
| L3. It's hard to predict my emotions from one moment to the next. | 2.13 (0.83) |  | 2.12 (0.79) |
| L4. I feel like I'm on an emotional roller coaster. | 2.17 (0.88) |  | 2.14 (0.86) |
| L5. My emotions can switch back and forth quickly. | 2.44 (0.83) |  | 2.38 (0.86) |
| L6. I can feel one way in one minute and feel totally different in the next. | 2.31 (0.87) |  | 2.26 (0.87) |
| L7. My emotions bounce around more than other people I know. | 2.09 (0.86) |  | 2.05 (0.84) |
| L8. My emotions don't change a lot. [R] | 2.64 (0.74) |  | 2.59 (0.71) |
| L9. My emotions about people or events change frequently. | 2.34 (0.78) |  | 2.30 (0.77) |
| L10. Others tell me my emotions change a great deal. | 1.89 (0.78) |  | 1.85 (0.78) |
| **Reactivity** (range = 1 to 4) | 2.46 (0.58) |  | 2.40 (0.50) |
| R1. My emotional reactions escalate quickly. | 2.59 (0.80) |  | 2.51 (0.81) |
| R2. I'm calm even when things don't go according to plan. [R] | 2.34 (0.78) |  | 2.31 (0.79) |
| R3. I don't react very strongly to things that happen. [R] | 2.65 (0.74) |  | 2.58 (0.71) |
| R4. It takes a lot to get a strong emotional reaction from me. [R] | 2.50 (0.94) |  | 2.42 (0.89) |
| R5. My emotions can go from neutral to intense quickly. | 2.37 (0.86) |  | 2.39 (0.79) |
| R6. When something stressful happens, my emotions get really strong. | 2.79 (0.84) |  | 2.73 (0.82) |
| R7. I've been described as having intense emotional reactions. | 2.15 (0.92) |  | 2.04 (0.84) |
| R8. I'm surprised by how strong my emotions are. | 2.31 (0.79) |  | 2.28 (0.71) |
| R9. My emotions get intense when things go wrong. | 2.53 (0.81) |  | 2.49 (0.80) |
| R10. My emotions get triggered easily. | 2.35 (0.81) |  | 2.25 (0.78) |
| **Consequences** (range = 1 to 4) | 2.18 (0.57) |  | 2.13 (0.53) |
| C1. My emotions cause problems or conflicts with other people. | 2.14 (0.81) |  | 2.08 (0.79) |
| C2. When I'm emotional, I don't make the best decisions. | 2.41 (0.81) |  | 2.41 (076) |
| C3. When I'm emotional, I do things that I later regret. | 2.10 (0.82) |  | 2.09 (0.79) |
| C4. My emotions run my life. | 1.99 (0.77) |  | 1.95 (0.76) |
| C5. My emotions have landed me in situations I didn't want to be in. | 2.26 (0.85) |  | 2.25 (0.83) |
| C6. It bothers me that I'm so emotional. | 2.21 (0.91) |  | 2.09 (0.86) |
| C7. My emotions rarely cause problems for me. [R] | 2.29 (0.82) |  | 2.22 (0.77) |
| C8. My emotions don't get the best of me. [R] | 2.29 (0.79) |  | 2.24 (0.77) |
| C9. My emotions rarely get me into trouble. [R] | 2.05 (0.75) |  | 2.02 (0.74) |
| C10. People get annoyed with me when I'm emotional. | 2.03 (0.80) |  | 1.98 (0.79) |
| *Note.* M = mean, SD = standard deviation. [R] indicates a reverse-scored item. | | | |

| **Table S2** |  |  |  |  |  |  |  |
| --- | --- | --- | --- | --- | --- | --- | --- |
| *Descriptive statistics for existing emotion regulation measures in Samples 3 and 4* | | | | | | | |
|  | Sample 3 | | |  | Sample 4 | | |
|  | *N* | *Mean* | *SD* |  | *N* | *Mean* | *SD* |
| DERS total score | 245 | 86.46 | 23.47 |  | 128 | 91.03 | 22.62 |
| DERS nonacceptance | 246 | 14.67 | 5.81 |  | 129 | 14.75 | 5.87 |
| DERS goals | 246 | 15.32 | 4.50 |  | 130 | 15.49 | 4.35 |
| DERS impulse | 246 | 11.48 | 4.85 |  | 129 | 12.60 | 5.12 |
| DERS awareness | 246 | 15.02 | 4.65 |  | 130 | 16.19 | 5.14 |
| DERS strategies | 245 | 18.50 | 7.14 |  | 130 | 19.49 | 7.43 |
| DERS clarity | 247 | 11.58 | 3.85 |  | 130 | 12.12 | 3.87 |
| DERS-18 total score | 246 | 41.47 | 12.01 |  | 128 | 43.34 | 11.97 |
| DERS-SF total score | 246 | 41.33 | 11.71 |  | 128 | 43.21 | 11.97 |
| DERS-16 total score | 245 | 37.80 | 12.77 |  | 128 | 39.24 | 12.99 |
| ERQ cognitive reappraisal | 237 | 26.55 | 7.26 |  | 127 | 25.80 | 7.24 |
| ERQ expressive suppression | 233 | 13.82 | 4.78 |  | 127 | 14.65 | 5.11 |
| PAI-BOR affective instability | 248 | 7.79 | 3.65 |  | 131 | 8.60 | 3.49 |
| PERS Negative total | 242 | 40.84 | 13.75 |  | 128 | 43.51 | 13.41 |
| Activation | 242 | 13.59 | 4.85 |  | 128 | 14.43 | 4.80 |
| Intensity | 242 | 14.12 | 5.09 |  | 128 | 15.10 | 4.78 |
| Duration | 243 | 13.12 | 4.73 |  | 128 | 13.98 | 4.78 |
| *Note.* M = mean, SD = standard deviation. DERS = Difficulties in Emotion Regulation Scale (Gratz & Roemer, 2004). ERQ = Emotion Regulation Questionnaire (Gross & John, 2003). PAI-BOR = Personality Assessment Inventory-Borderline features scale (Morey, 1991). PERS = Perth Emotional Reactivity Scale (Becerra et al., 2019). | | | | | | | |

| **Table S3** |  |  |  |  |  |  |  |
| --- | --- | --- | --- | --- | --- | --- | --- |
| *Descriptive statistics for theoretically relevant external criteria in Samples 3 and 4* | | | | | | | |
|  | Sample 3 | | |  | Sample 4 | | |
|  | *N* | *Mean* | *SD* |  | *N* | *Mean* | *SD* |
| PAI-BOR total score | 247 | 37.87 | 11.52 |  | 131 | 39.78 | 10.63 |
| PAI-BOR identity disturbance | 247 | 12.34 | 3.75 |  | 131 | 12.24 | 3.63 |
| PAI-BOR negative relationships | 248 | 9.47 | 3.66 |  | 131 | 9.88 | 3.52 |
| PAI-BOR self-harm | 248 | 8.33 | 3.34 |  | 131 | 9.05 | 3.09 |
| UPPS-P negative urgency | 247 | 2.32 | 0.51 |  | 131 | 2.42 | 0.47 |
| UPPS-P (lack of) premeditation | 247 | 1.98 | 0.44 |  | 131 | 2.11 | 0.43 |
| UPPS-P (lack of) perseverance | 247 | 1.94 | 0.54 |  | 131 | 2.11 | 0.45 |
| UPPS-P sensation seeking | 247 | 2.61 | 0.54 |  | 131 | 2.74 | 0.49 |
| UPPS-P positive urgency | 247 | 1.96 | 0.50 |  | 131 | 2.13 | 0.52 |
| AAQ-II | 245 | 26.40 | 9.75 |  | 130 | 28.81 | 11.59 |
| AUDIT | 246 | 6.33 | 5.94 |  | 129 | 6.76 | 6.70 |
| DUDIT | 243 | 3.19 | 5.73 |  | 129 | 3.49 | 6.50 |
| DSHI yes/no | 245 | 0.29 | 0.45 |  | 129 | 0.30 | 0.46 |
| DSHI frequency | 245 | 13.33 | 76.89 |  | 129 | 3.14 | 12.00 |
| DSHI number of methods | 245 | 0.84 | 1.73 |  | 129 | 0.78 | 1.58 |
| RRS | 242 | 43.14 | 15.13 |  | 127 | 45.66 | 16.55 |
| PID-5-BF negative affectivity | 246 | 1.14 | 0.73 |  | 130 | 1.15 | 0.73 |
| PID-5-BF detachment | 246 | 0.59 | 0.54 |  | 130 | 0.66 | 0.58 |
| PID-5-BF antagonism | 246 | 0.46 | 0.52 |  | 130 | 0.62 | 0.59 |
| PID-5-BF disinhibition | 246 | 0.44 | 0.55 |  | 130 | 0.57 | 0.63 |
| PID-5-BF psychoticism | 246 | 0.63 | 0.63 |  | 130 | 0.74 | 0.67 |
| FFMQ | 238 | 125.08 | 17.23 |  | 127 | 121.24 | 13.26 |
| PHQ-9 | 242 | 7.46 | 6.10 |  | 128 | 7.47 | 6.93 |
| GAD-7 | 243 | 6.11 | 5.46 |  | 129 | 6.33 | 6.11 |
| In treatment | 249 | 0.23 | 0.42 |  | 132 | 0.23 | 0.43 |
| *Note.* M = mean, SD = standard deviation. PAI-BOR = Personality Assessment Inventory-Borderline features scale (Morey, 1991). UPPS-P = Urgency, Premeditation, Perseverance, Sensation Seeking, and Positive Urgency Impulsive Behavior Scale (Lynam et al., 2006). AAQ-II = Acceptance and Action Questionnaire-II (Bond et al., 2011). AUDIT = Alcohol Use Disorders Identification Test (Saunders et al., 1993). DUDIT = Drug Use Disorders Identification Test (Berman et al., 2005). DSHI = Deliberate Self-Harm Inventory (Gratz, 2001). RRS = Ruminative Response Scale (Treynor et al., 2003). PID-5-BF = Personality Inventory for DSM-5 Brief Form (Anderson et al., 2018). FFMQ = Five Facet Mindfulness Questionnaire (Baer et al., 2006). PHQ-9 = Patient Health Questionnaire Depression Module (Kroenke et al., 2001). GAD-7 = Generalized Anxiety Disorder scale (Spitzer et al., 2006). | | | | | | | |

| **Table S4** |  |  |  |
| --- | --- | --- | --- |
| *Descriptive statistics for other psychopathology measures in Sample 5* | | | |
|  | *N* | *Mean* | *SD* |
| IDAS-II general depression | 210 | 42.78 | 13.57 |
| ASI total score | 209 | 37.37 | 13.17 |
| ESI total score | 183 | 99.36 | 49.20 |
| ESI general disinhibition | 207 | 10.00 | 7.00 |
| ESI callous-aggression | 206 | 8.67 | 7.19 |
| ESI substance abuse | 202 | 22.80 | 10.20 |
| *Note.* IDAS-II = Inventory of Depression and Anxiety Symptoms-II (Watson et al., 2012). ASI = Anxiety Sensitivity Index (Rodriguez et al., 2004). ESI = Externalizing Symptom Inventory Brief Form (Patrick et al., 2016). | | | |

| **Table S5** |  |  |  |  |  |
| --- | --- | --- | --- | --- | --- |
| *Zero-order correlations among the DERS and PERS with primary convergent measures in Samples 3 and 4 for comparison with the performance of the BEDS* | | | | | |
|  | DERS Total | DERS-18 | DERS-SF | DERS-16 | PERS Neg Total |
| DERS total score | -- | **.98 /.98** | **.98 /.98** | **.95 /.93** | **.57 /.50** |
| DERS nonaccept | **.82 /.81** | **.81 /.83** | **.81 /.83** | **.81 /.84** | **.42 /.43** |
| DERS goals | **.68 /.61** | **.67 /.61** | **.67 /.63** | **.74 /.70** | **.58 /.46** |
| DERS impluse | **.81 /.75** | **.81 /.74** | **.80 /.74** | **.80 /.74** | **.40 /.39** |
| DERS awareness | **.52 /.40** | **.48 /**.31 | **.49 /.32** | **.26 /**.07 | .12 **/**-.05 |
| DERS strategies | **.90 /.90** | **.85 /.87** | **.85 /.88** | **.93 /.92** | **.60 /.61** |
| DERS clarity | **.76 /.67** | **.76 /.68** | **.77 /.66** | **.65 /.51** | **.34 /**.15 |
| DERS-18 total score | **.98 /.98** | -- | **.99 /.98** | **.94 /.94** | **.54 /.49** |
| DERS-SF total score | **.98 /.98** | **.99 /.98** | -- | **.94 /.94** | **.54 /.50** |
| DERS-16 total score | **.95 /.93** | **.94 /.94** | **.94 /.94** | -- | **.59 /.58** |
| ERQ cognitive reappraisal | **-.45 /-.37** | **-.39 /-.33** | **-.41 /-.33** | **-.37 /**-.24 | **-.32 /**-.02 |
| ERQ expressive suppression | **.30 /**.14 | **.29 /**.13 | **.30 /**.12 | .21 **/**.12 | .09 **/**.06 |
| PAI-BOR affective instability | **.71 /.63** | **.68 /.62** | **.69 /.61** | **.72 /.60** | **.59 /.53** |
| PERS Negative total | **.57 /.50** | **.54 /.49** | **.54 /.50** | **.59 /.58** | -- |
| Activation | **.54 /.49** | **.52 /.48** | **.52 /.48** | **.55 /.55** | **.94 /.95** |
| Intensity | **.50 /.44** | **.49 /.44** | **.48 /.44** | **.55 /.53** | **.94 /.93** |
| Duration | **.56 /.48** | **.52 /.46** | **.52 /.47** | **.57 /.55** | **.94 /.92** |
| *Note.* DERS = Difficulties in Emotion Regulation Scale (Gratz & Roemer, 2004). ERQ = Emotion Regulation Questionnaire (Gross & John, 2003). PAI-BOR = Personality Assessment Inventory-Borderline features scale (Morey, 1991). PERS = Perth Emotional Reactivity Scale (Becerra et al., 2019). Correlations significant at Bonferroni corrected *p* < .00025 are **bolded**. Missing data were handled using pairwise deletion, resulting in *n*s for the correlations in this table ranging from 230 to 246 for Sample 3 and 125 to 128 for Sample 4. Correlations for Sample 3 appear to the left of the "/" and those for Sample 4 appear to the right. | | | | | |

| **Table S6** |  |  |  |  |  |
| --- | --- | --- | --- | --- | --- |
| *Zero-order correlations among the DERS and PERS with associated constructs in Samples 3 and 4 for comparison with the performance of the BEDS* | | | | | |
|  | DERS Total | DERS-18 | DERS-SF | DERS-16 | PERS Neg Total |
| PAI-BOR total score | **.73 /.71** | **.72 /.70** | **.72 /.69** | **.74 /.70** | **.62 /.53** |
| PAI-BOR identity disturbance | **.65 /.66** | **.61 /.65** | **.62 /.65** | **.65 /.67** | **.60 /.51** |
| PAI-BOR negative relationships | **.51 /.58** | **.51 /.55** | **.50 /.56** | **.51 /.59** | **.49 /.49** |
| PAI-BOR self-harm | **.48 /**.29 | **.52 /**.29 | **.52 /**.27 | **.48 /**.24 | **.25 /**.07 |
| UPPS-P negative urgency | **.64 /.49** | **.64 /.45** | **.64 /.45** | **.64 /.42** | **.46 /.34** |
| UPPS-P (lack of) premeditation | .18 **/**.10 | .19 **/**.11 | .19 **/**.11 | .09 **/**.07 | -.02 /-.13 |
| UPPS-P (lack of) perseverance | **.53 /**.30 | **.52 /**.29 | **.54 /**.28 | **.48 /**.22 | **.28 /**-.03 |
| UPPS-P sensation seeking | -.07 **/**-.25 | -.03 **/**-.27 | -.03 **/**-.26 | -.04 **/**-.28 | -.12 **/**-.17 |
| UPPS-P positive urgency | **.50 /.45** | **.53 /.41** | **.53 /.41** | **.48 /**.31 | .20 **/**.10 |
| AAQ-II | **.70 /.73** | **.66 /.71** | **.67 /.73** | **.71 /.73** | **.67 /.56** |
| AUDIT | **.23 /**.09 | **.25 /**.08 | **.24 /**.08 | **.24 /**.04 | .15 /-.02 |
| DUDIT | .20 **/**.15 | .23 **/**.13 | .23 **/**.13 | **.25 /**.12 | .19 /.04 |
| DSHI yes/no | **.39 /.33** | **.36 /**.30 | **.37 /**.30 | **.41 /**.29 | **.35 /**.15 |
| DSHI frequency | .16 **/**.16 | .18 **/**.09 | .16 **/**.14 | .16 **/**.12 | .15 **/**.09 |
| DSHI number of methods | **.39 /**.32 | **.38 /**.28 | **.39 /**.28 | **.39 /**.26 | **.29 /**.16 |
| RRS | **.61 /.57** | **.60 /.55** | **.61 /.56** | **.65 /.60** | **.67 /.62** |
| PID-5-BF negative affectivity | **.62 /.61** | **.58 /.60** | **.58 /.61** | **.65 /.63** | **.66 /.62** |
| PID-5-BF detachment | **.55 /.54** | **.57 /.55** | **.57 /.55** | **.51 /.50** | **.42 /**.30 |
| PID-5-BF antagonism | **.34 /.42** | **.39 /.45** | **.39 /.44** | **.35 /.43** | .19 **/**.24 |
| PID-5-BF disinhibition | **.43 /.36** | **.48 /.36** | **.47 /.36** | **.42 /.33** | **.25 /**.14 |
| PID-5-BF psychoticism | **.58 /.46** | **.61 /.46** | **.60 /.48** | **.59 /.43** | **.39 /**.27 |
| FFMQ | **-.73 /-.68** | **-.68 /-.63** | **-.69 /-.62** | **-.63 /-.51** | **-.45 /**-.27 |
| PHQ-9 | **.54 /.57** | **.53 /.54** | **.55 /.55** | **.57 /.58** | **.54 /.55** |
| GAD-7 | **.50 /.57** | **.46 /.57** | **.47 /.56** | **.55 /.60** | **.59 /.63** |
| In treatment | **.28 /**.17 | **.26 /**.14 | **.26 /**.14 | **.29 /**.14 | **.27 /**.17 |
| *Note.* DERS = Difficulties in Emotion Regulation Scale (Gratz & Roemer, 2004). PERS = Perth Emotional Reactivity Scale (Becerra et al., 2019). PAI-BOR = Personality Assessment Inventory-Borderline features scale (Morey, 1991). UPPS-P = Urgency, Premeditation, Perseverance, Sensation Seeking, and Positive Urgency Impulsive Behavior Scale (Lynam et al., 2006). AAQ-II = Acceptance and Action Questionnaire-II (Bond et al., 2011). AUDIT = Alcohol Use Disorders Identification Test (Saunders et al., 1993). DUDIT = Drug Use Disorders Identification Test (Berman et al., 2005). DSHI = Deliberate Self-Harm Inventory (Gratz, 2001). RRS = Ruminative Response Scale (Treynor et al., 2003). PID-5-BF = Personality Inventory for DSM-5 Brief Form (Anderson et al., 2018). FFMQ = Five Facet Mindfulness Questionnaire (Baer et al., 2006). PHQ-9 = Patient Health Questionnaire Depression Module (Kroenke et al., 2001). GAD-7 = Generalized Anxiety Disorder scale (Spitzer et al., 2006). Correlations significant at Bonferroni corrected *p* < .00025 are **bolded**. Missing data were handled using pairwise deletion, resulting in *n*s for the correlations in this table ranging from 237 to 246 for Sample 3 and 126 to 128 for Sample 4. Correlations for Sample 3 appear to the left of the "/" and those for Sample 4 appear to the right. | | | | | |
